# Metabolic-Protective Strategies for Antipsychotic-Associated Weight Gain in Children and Adolescents: A Systematic Review

**DOI:** 10.7759/cureus.114599

**Published:** 2026-08-16

**Authors:** Ali J Alhagawy, Ibrahim J Alhiqwi, Hamad A Majrabi, Ayoub H Salami, Mohammed S Aldawsari, Mohammed A Shok, Atyaf A Kariri, Ahlam M Muharraq, Raghad K Juraybi, Abdullah M Alghamdi, Abdulrahman M Alshaiban

**Affiliations:** 1 Diabetic Center, Jazan Health Cluster, Jazan, SAU; 2 Psychiatry Department, Eradah Hospital, Jazan, SAU; 3 Faculty of Medicine, Jazan University, Jazan, SAU; 4 Faculty of Medicine, Tabuk University, Tabuk, SAU

**Keywords:** antipsychotic-associated weight gain, body mass index z-score, children and adolescents, metabolic dysfunction, metformin, obesity prevention, pediatric psychopharmacology, randomized controlled trials, second-generation antipsychotics, systematic review

## Abstract

Second-generation antipsychotics are effective for a range of pediatric psychiatric and behavioral conditions but are commonly associated with weight gain and metabolic dysfunction. Metformin is the most extensively studied pharmacological strategy for preventing or treating this adverse effect; however, the randomized evidence base has evolved substantially with the publication of recent large trials.

This study aimed to systematically review and meta-analyze randomized controlled trials of metformin and other metabolic-protective strategies, including topiramate, melatonin, and antipsychotic switching, for preventing or treating antipsychotic-associated weight gain in children and adolescents. PubMed/MEDLINE, Scopus, Web of Science, and Cochrane Central Register of Controlled Trials were searched for records published between January 2000 and June 2026. Randomized controlled trials enrolling participants aged 19 years or younger who were receiving a second-generation antipsychotic were eligible. Risk of bias was assessed using the Cochrane Risk of Bias 2 tool. Random-effects meta-analyses using restricted maximum likelihood estimation with the Hartung-Knapp adjustment pooled mean differences and risk ratios when at least two trials contributed comparable data; prevention and treatment indications were analyzed separately.

Seven unique randomized controlled trials reported across eight publications met eligibility criteria, including 1,871 participants: five trials evaluated metformin, one evaluated topiramate, and one evaluated melatonin. The pooled effect of metformin on body mass index (BMI) z-score, the primary outcome, favored treatment at all time windows but was not statistically significant (short-term, three trials: mean difference [MD] = -0.093, 95% confidence interval [CI] -0.250 to 0.063; medium-term, two trials: MD = -0.090, 95% CI -0.346 to 0.166; long-term, one trial: MD = -0.07, 95% CI -0.145 to 0.005). Short-term body weight was significantly reduced with metformin (three trials: MD = -2.30 kg, 95% CI -4.28 to -0.32). Metabolic laboratory outcomes were imprecise and inconsistent in direction, and diarrhea was numerically more frequent with metformin. Evidence for topiramate, melatonin, and antipsychotic switching was limited to single trials or trial arms. No included trial was rated as having an overall low risk of bias.

Metformin has the largest randomized pediatric evidence base among metabolic-protective strategies and consistently attenuated weight gain compared with control; however, its pooled effect on the primary outcome did not reach statistical significance at any time window, and metabolic benefits could not be confirmed. Decisions regarding metabolic-protective strategies should be individualized rather than universally applied, and larger, longer, standardized pediatric trials are needed.

## Introduction and background

Second-generation antipsychotics are widely used to manage serious pediatric psychiatric and behavioral conditions, including schizophrenia-spectrum disorders, bipolar disorder, and autism-associated irritability [1-3]. Although effective for controlling psychosis, mood symptoms, and severe aggression, their use is frequently prolonged because these disorders are often chronic or recurrent. This sustained exposure during childhood and adolescence is associated with clinically important adverse effects, particularly weight gain and metabolic dysfunction [4-8].

Antipsychotic-associated weight gain can occur rapidly in youth, with greater vulnerability observed among children, younger patients, and those receiving antipsychotics for the first time. The risk varies across agents and extends beyond increased body weight to abnormalities in body mass index (BMI), central adiposity, insulin sensitivity, glucose regulation, lipid profiles, and blood pressure. Because children and adolescents are still developing, pediatric studies commonly use age- and sex-adjusted measures such as BMI z-score rather than adult weight thresholds [2-7].

The consequences of antipsychotic-associated weight gain may persist beyond treatment and increase long-term cardiometabolic risk. Weight-related distress can also affect self-esteem, social functioning, and adherence to psychiatric treatment [6-10]. Discontinuing or reducing an effective antipsychotic may increase the risk of psychiatric relapse, creating a need for strategies that preserve psychiatric stability while limiting metabolic harm [1,11].

Current approaches include metabolic monitoring, lifestyle interventions, dose optimization, switching to lower-risk antipsychotics, and adjunctive pharmacological treatments. However, lifestyle interventions have shown limited and inconsistent effects in this population, while dose reduction or switching may compromise symptom control [10-13]. These limitations have increased interest in pharmacological strategies that can be added without interrupting effective antipsychotic therapy.

Metformin, a biguanide insulin-sensitizing medication primarily used for type 2 diabetes, is the most extensively studied adjunctive treatment for antipsychotic-associated weight gain. Its potential benefits are related to improved insulin sensitivity, reduced hepatic glucose production, and possible effects on appetite regulation and metabolic signaling [2-7]. Pediatric randomized trials have evaluated metformin both as prevention, initiated near the start of antipsychotic treatment, and as treatment after significant weight gain has occurred. However, studies differ substantially in participant characteristics, antipsychotic exposure, intervention timing, follow-up duration, and outcome measures, limiting conclusions regarding its overall effectiveness.

Other metabolic-protective strategies, including topiramate, melatonin, and antipsychotic switching, have also been investigated but have substantially smaller pediatric evidence bases. These approaches involve distinct mechanisms and clinical considerations and should not be considered interchangeable with metformin [2-8]. In particular, evidence outside metformin remains limited, with few randomized trials and uncertainty regarding comparative effectiveness, safety, and effects on psychiatric outcomes.

Previous studies have provided important evidence but were limited by the number and size of available trials, inclusion of non-randomized studies, and the absence of newer large randomized trials. The evidence base has since expanded, requiring an updated synthesis that focuses exclusively on randomized controlled trials, distinguishes prevention from treatment strategies, accounts for differences between interventions, and evaluates both metabolic outcomes and clinical safety. Therefore, this systematic review and meta-analysis aimed to assess the efficacy, metabolic effects, safety, and acceptability of metformin and other randomized metabolic-protective strategies for preventing or treating antipsychotic-associated weight gain in children and adolescents.

## Review

Methods

Search and Study Selection

This systematic review and meta-analysis was conducted according to the Preferred Reporting Items for Systematic Reviews and Meta-Analyses (PRISMA) 2020 statement [14]. The review protocol was not prospectively registered.

Eligible studies were randomized controlled trials enrolling children and adolescents aged 19 years or younger who were receiving, initiating, or planning to initiate a second-generation antipsychotic. Studies with mixed-age populations were included when pediatric data could be extracted separately. Eligible interventions included metformin and other metabolic-protective strategies, including topiramate, melatonin, and antipsychotic switching, intended to prevent or treat antipsychotic-associated weight gain or metabolic abnormalities. Eligible comparators included placebo, lifestyle intervention, continuation of the existing antipsychotic with supportive care, or another active intervention. Outcomes included anthropometric measures (primary outcome: body mass index [BMI] z-score; secondary outcomes: body weight, BMI, weight-related indices), metabolic measures (glucose, insulin, homeostatic model assessment of insulin resistance [HOMA-IR], glycated hemoglobin [HbA1c], lipid parameters, and blood pressure), and safety outcomes (adverse events, treatment discontinuation, and psychiatric stability).

Observational studies, non-randomized comparisons, single-arm studies, case series, reviews, protocols without results, adult-only studies, and pediatric obesity studies unrelated to antipsychotic exposure were excluded. PubMed/MEDLINE, Scopus, Web of Science Core Collection, and the Cochrane Central Register of Controlled Trials were searched for studies published from January 2000 to June 2026 using terms related to antipsychotic treatment, pediatric populations, metabolic outcomes, and eligible interventions. The search strategy combined controlled vocabulary terms and free-text keywords related to second-generation antipsychotics (e.g., atypical antipsychotic, risperidone, olanzapine), pediatric populations (child, adolescent, youth), metabolic outcomes (weight gain, obesity, BMI, insulin resistance, metabolic abnormalities), and eligible interventions (metformin, topiramate, melatonin, antipsychotic switching). Search terms were adapted for each database.

Reference lists, citation tracking, and trial registries were reviewed to identify additional studies and companion publications. Two reviewers independently screened records and full texts, with disagreements resolved through discussion or third-reviewer adjudication. Reports from the same randomized sample were linked, and the most complete publication was used as the primary data source.

Data Extraction

Data were extracted into a standardized spreadsheet and verified against the original reports and supplementary materials. Extracted data included study design, setting, duration, participant characteristics, diagnosis, antipsychotic exposure, intervention details, comparator conditions, and outcome data. Extracted outcome information included means, measures of variability, confidence intervals, adjusted estimates, and event counts. Data discrepancies and reporting limitations were recorded.

Follow-up duration was categorized as short term (approximately 12-16 weeks), medium term (approximately 24 weeks or 6 months), or long term (>6 months). Only one time point per study was included in each pooled analysis. Trials were classified as prevention studies when the intervention was initiated near the start of antipsychotic treatment and as treatment studies when the intervention was started after clinically significant weight gain or metabolic deterioration.

Quality Assessment

Risk of bias was assessed at the outcome level using the revised Cochrane Risk of Bias 2 (RoB 2) tool for randomized trials [15]. Domains included bias arising from randomization, deviations from intended interventions, missing outcome data, outcome measurement, and selective reporting. Each domain was rated as low risk, some concerns, or high risk, with an overall judgment based on the RoB 2 algorithm. Particular consideration was given to attrition, post-randomization exclusions, open-label designs, and selective reporting.

Data Synthesis

Continuous outcomes were pooled as mean differences with 95% confidence intervals, while dichotomous outcomes were summarized as risk ratios with 95% confidence intervals. Adjusted estimates were included when appropriate variance measures were available. Random-effects meta-analysis models were used to account for clinical and methodological heterogeneity, with between-study variance estimated using restricted maximum likelihood. Heterogeneity was assessed using tau-squared, Cochran’s Q, and I-squared statistics. Because of the small number of studies contributing to each analysis, confidence intervals were calculated using the Hartung-Knapp adjustment, with conventional random-effects estimates examined in sensitivity analyses.

Metformin studies were analyzed separately from topiramate, melatonin, and antipsychotic switching studies. Prevention and treatment studies were also analyzed separately. For the three-arm IMPACT trial, intervention groups were compared separately with the shared control group to avoid double-counting. Sensitivity analyses excluded studies at high risk of bias and studies requiring reconstructed variance estimates. Leave-one-out analyses were performed when at least three studies contributed to an outcome. Small-study effects were not assessed because no analysis included 10 or more comparable studies. All analyses were conducted in R using the meta and metafor packages.

Results

Study Selection

**Figure 1:**
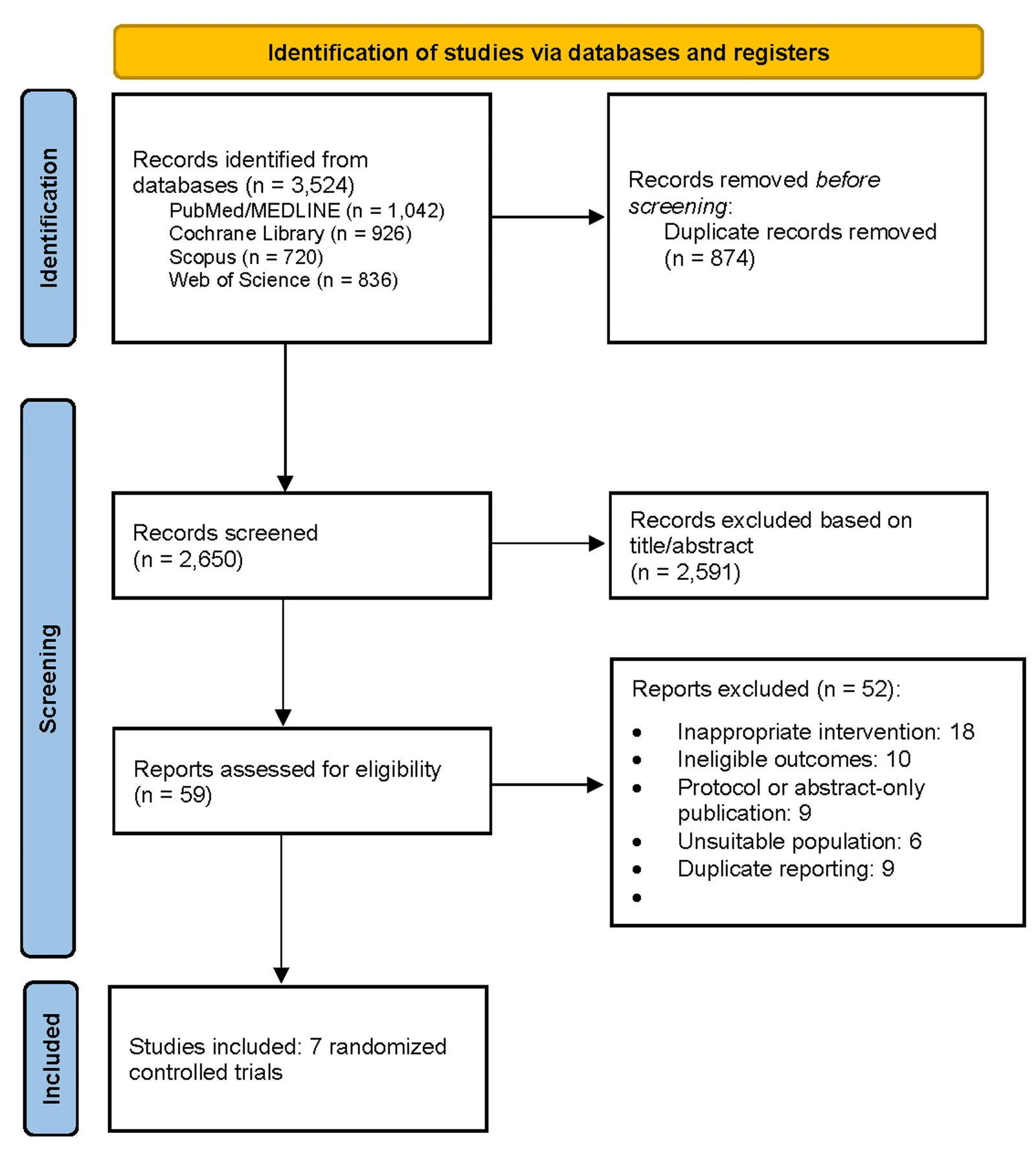
PRISMA flow diagram depicting the study selection process for the systematic review PRISMA (Preferred Reporting Items for Systematic Reviews and Meta-Analyses) flow diagram detailing the study selection process for this systematic review. Following the identification of records through database searching and other sources, duplicates were removed, and the remaining records were screened. Full-text articles were assessed for eligibility, with exclusions documented along with reasons. Studies meeting the inclusion criteria were included in the final synthesis.

The database search identified 3,524 records. After removing 874 duplicates, 2,650 records were screened, and 59 full-text articles were assessed for eligibility. Seven unique RCTs, reported across eight publications, met the inclusion criteria [1-8]. Companion reports describing the same trial were treated as a single study. In the three-arm IMPACT trial, the metformin and antipsychotic-switch groups were each compared separately with the shared control group to avoid double-counting (Figure 1).

Study Characteristics

The seven RCTs included 1,871 children and adolescents (sample size range, 30-1,565). Five trials evaluated metformin [1-4,8], one evaluated topiramate [5], and one evaluated melatonin [6,7]. One metformin trial investigated prevention of antipsychotic-associated weight gain in antipsychotic-naïve participants [1], whereas the remaining metformin trials evaluated treatment of established overweight or obesity [2-4,8]. Follow-up ranged from 12 weeks to 24 months. Five trials were double-blind and placebo-controlled [1-3,5-7], whereas the IMPACT and MOBILITY trials were open-label pragmatic studies [4,8].

Participants had a mean baseline age of approximately 9-15 years. Antipsychotics included risperidone, olanzapine, quetiapine, and aripiprazole. Baseline body mass index (BMI) ranged from normal weight in prevention studies to overweight or obesity in treatment studies (Table 1).

**Table 1:** Characteristics and intervention details of included randomized controlled trials RCT, randomized controlled trial; NCT, ClinicalTrials.gov registration number; IRCT, Iranian Registry of Clinical Trials; SGA, second-generation antipsychotic; DMDD, disruptive mood dysregulation disorder; NOS, not otherwise specified; LIFE, lifestyle intervention consisting of healthy eating and physical activity education. Study identifiers correspond to the numbered reference list. Companion publications from the same randomized trial are presented under a single study identifier. Doses and titration schedules are reported as described in the original publications. Registration information is reported when available; “not reported” indicates that registration details were unavailable in the published report.

Study ID	Country / Setting	Recruitment period / Registration	Design	Diagnosis	Age criterion	Antipsychotic	Intervention	Comparator	Duration
Arman [1]	Iran — Child and Adolescent Psychiatric Consultation Center, Isfahan University of Medical Sciences; single center	October 2006–October 2007; Registration: Not reported	Double-blind, placebo-controlled RCT	Schizophrenia only (analyzed sample); schizoaffective enrollees (n=2) were among the 17 excluded	<20 years	Risperidone 2–6 mg/day in Methods; abstract states 6 mg	Metformin 500 mg each morning for 1 week, then 500 mg twice daily	Identical placebo	12 weeks
Klein [2]	United States — Cincinnati Children’s Hospital Medical Center and University of Cincinnati; clinical research center	Not reported; Registration: Not reported	Double-blind, placebo-controlled RCT	Heterogeneous psychiatric diagnoses: bipolar disorder, attention disorders, schizophrenia, oppositional defiant disorder, autism/Asperger syndrome, Tourette syndrome, schizoaffective disorder, and depression	10–17 years	Olanzapine, risperidone, or quetiapine; stable regimen	Metformin 500 mg with evening meal during week 1; 500 mg twice daily during week 2; 850 mg twice daily for the remaining 14 weeks	Identical placebo on matched schedule; both groups received individualized dietary counseling	16 weeks
Anagnostou [3]	Canada and United States — Four academic centers: Toronto, Columbus, Pittsburgh, and Nashville	26 April 2013–24 June 2015; Registration: NCT01825798	Double-blind, placebo-controlled multicenter RCT	Autism spectrum disorder with irritability/agitation treated with an atypical antipsychotic	6–17 years 4 months	Risperidone, aripiprazole, quetiapine, olanzapine, ziprasidone, or iloperidone at a stable dose	Liquid metformin: titrated to 500 mg twice daily for ages 6–9 years and 850 mg twice daily for ages 10–17 years	Matching liquid placebo with identical titration; both groups received brief diet and exercise counseling	16 weeks
Correll [4]	United States — Four universities: Zucker/Hillside, Johns Hopkins, University of Maryland, and University of North Carolina	October 2009–October 2013; Registration: Protocol published: Reeves et al. 2013	Open-label, three-arm, randomized comparative-effectiveness trial	Schizophrenia spectrum disorder, bipolar spectrum disorder, or major depression with psychotic features	8–19 years	Stable baseline second-generation antipsychotic; switch arm changed to aripiprazole or, when needed, perphenazine or molindone	Weight-based metformin titration to 1000–2000 mg/day plus multivitamin and healthy lifestyle education	Continued baseline antipsychotic plus healthy lifestyle education; separate switch arm also received healthy lifestyle education	24 weeks
DelBello [5]	United States — University of Cincinnati and Cincinnati Children’s Hospital; inpatient units and outpatient clinics	2007–2010; Registration: NCT00394095	Double-blind, placebo-controlled pilot RCT	Bipolar I disorder, current manic or mixed episode	10–18 years	Olanzapine initiated at 5–10 mg/day; flexible titration to a maximum of 20 mg by day 21	Topiramate 25 mg twice daily initially; titrated over 4 weeks to 100 mg twice daily; increased to up to 200 mg twice daily by week 6 if tolerated	Matched placebo with the same titration schedule	12 weeks
Mostafavi [6,7]	Iran — Two psychiatric outpatient clinics using the same raters; Tehran University of Medical Sciences/Roozbeh Hospital	Not reported; Registration: IRCT201208071556N44	Double-blind, placebo-controlled RCT (two companion reports)	Bipolar I disorder, first diagnosis/current mania; major depressive disorder excluded	11–17 years	Olanzapine 5–10 mg/day initiated in both groups; lithium carbonate also initiated and titrated to therapeutic serum concentration	Melatonin 3 mg/day added to olanzapine and lithium	Matched placebo added to olanzapine and lithium	12 weeks
DelBello [8] (MOBILITY)	United States — 64 sites in 10 states (California, Connecticut, Kentucky, Maryland, Massachusetts, New Jersey, New York, Ohio, Pennsylvania, and Texas): 39 community mental-health clinics and 25 academic health centers	Enrollment (first consent) began 5 November 2015; recruitment (last consent) closed 10 February 2022; overall data collection continued through 30 December 2022; Registration: NCT02515773	Open-label, pragmatic, multisite randomized trial	Bipolar I, bipolar II, cyclothymic, specified/unspecified bipolar-related disorder, disruptive mood dysregulation disorder (DMDD), or mood disorder NOS	8–19 years	New or ongoing oral second-generation antipsychotic; most commonly aripiprazole, risperidone, and quetiapine	Metformin plus LIFE; recommended initiation at 500 mg daily with titration to a maximum of 1000 mg twice daily, flexibly prescribed in usual care	LIFE alone: brief video and written healthy eating and physical activity education; treatment crossover permitted	Up to 24 months

Risk of Bias

Using the Cochrane Risk of Bias 2 tool, four trials were judged to have a high risk of bias owing primarily to attrition, missing outcome data, or selective reporting [1,5-8]. The remaining three trials were rated as having some concerns [2-4]. No trial was judged to have a low overall risk of bias (Table 2).

**Table 2:** Risk-of-bias assessment of included randomized controlled trials using the Cochrane RoB 2 tool RoB 2, revised Cochrane Risk of Bias 2 tool; D1, bias arising from the randomization process; D2, bias due to deviations from intended interventions; D3, bias due to missing outcome data; D4, bias in measurement of the outcome; D5, bias in selection of the reported result. Overall risk-of-bias judgments were assigned according to the RoB 2 algorithm and categorized as low risk, some concerns, or high risk. Study identifiers correspond to the numbered reference list. Companion publications from the same randomized trial are presented under a single study identifier.

Study ID	D1: Bias due to randomization	D2: Bias due to deviations from intended interventions	D3: Bias due to missing outcome data	D4: Bias due to measurement of the outcome	D5: Bias due to selection of the reported result	Overall risk of bias
Arman [1]	Some concerns	High risk	High risk	Low risk	High risk	High risk
Klein [2]	Some concerns	Low risk	Some concerns	Low risk	Some concerns	Some concerns
Anagnostou [3]	Low risk	Low risk	Some concerns	Low risk	Low risk	Some concerns
Correll [4] (IMPACT)	Low risk	Some concerns	Some concerns	Low risk	Some concerns	Some concerns
DelBello [5] (topiramate)	Some concerns	Some concerns	High risk	Low risk	Some concerns	High risk
Mostafavi [6,7]	Low risk	Some concerns	High risk	Low risk	Some concerns	High risk
DelBello [8] (MOBILITY)	Low risk	Some concerns	High risk	Low risk	Low risk	High risk

Effects of Metformin

Four RCTs evaluated metformin for treatment of established antipsychotic-associated weight gain [2-4,8]. Analyses were performed separately for short-term (12-16 weeks), medium-term (approximately 24 weeks or 6 months), and long-term (>6 months) follow-up.

Body Mass Index Z-score

**Figure 2:**
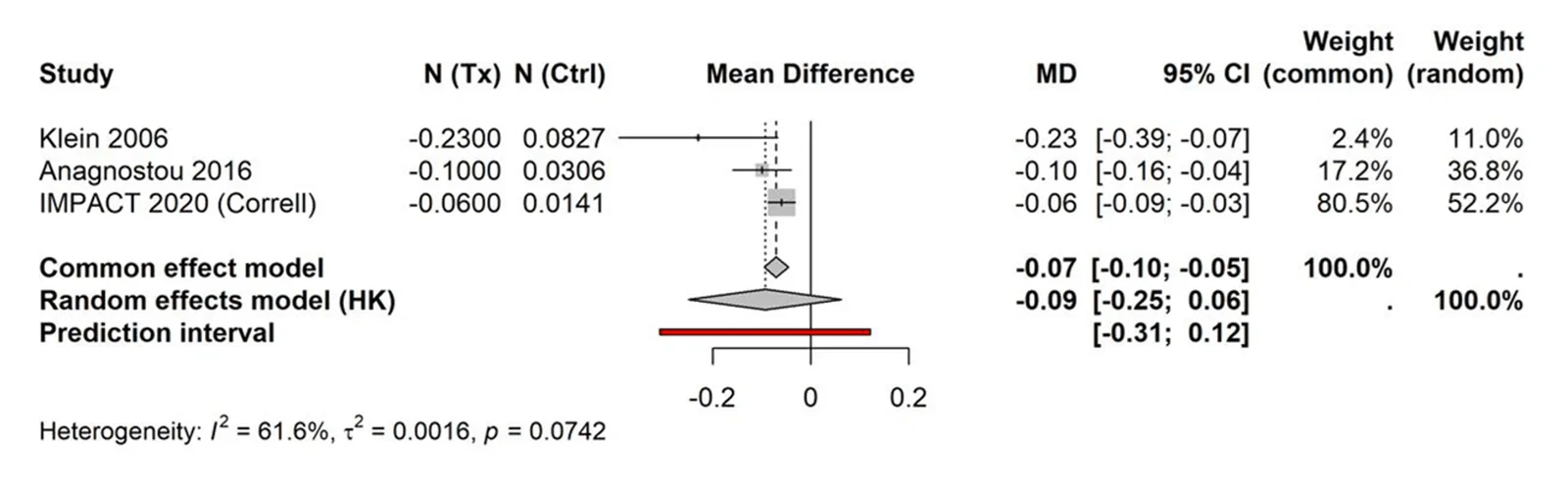
Forest plot of the effect of metformin versus control on BMI z-score in children and adolescents receiving second-generation antipsychotics Forest plot showing pooled mean differences (MDs) with 95% confidence intervals (CIs) for the effect of metformin compared with control on BMI z-score at short-term follow-up (approximately 12–16 weeks). Negative values indicate lower BMI z-scores in the metformin group. Squares represent study-specific effect estimates, horizontal lines represent 95% CIs, and the diamond represents the pooled random-effects estimate. Statistical pooling was performed using a random-effects model with restricted maximum likelihood estimation and Hartung-Knapp adjustment [2-4].

Body mass index z-score (BMI z-score) was the primary outcome. In the short term, three trials (n=181) showed a pooled mean difference (MD) of −0.093 (95% confidence interval [CI], −0.250 to 0.063; I²=61.6%) favoring metformin, although the result was not statistically significant (Figure 2). Medium-term analysis of two trials (n=1,343) also favored metformin (MD, −0.090; 95% CI, −0.346 to 0.166; I²=15.2%) without reaching statistical significance. Long-term evidence was limited to a single trial, which likewise favored metformin (MD, −0.07; 95% CI, −0.145 to 0.005). The forest plots of the medium-term and long-term analyses can be found in the Appendix. 

Body Weight and Anthropometric Outcomes

Three short-term trials demonstrated a significant reduction in body weight with metformin (MD, −2.30 kg; 95% CI, −4.28 to −0.32; I²=19.0%). Raw BMI and weight z-score showed no significant pooled differences. Other anthropometric outcomes, including waist circumference, BMI percentile, and weight percentile, were reported by single trials only.

Metabolic Outcomes

**Figure 3:**
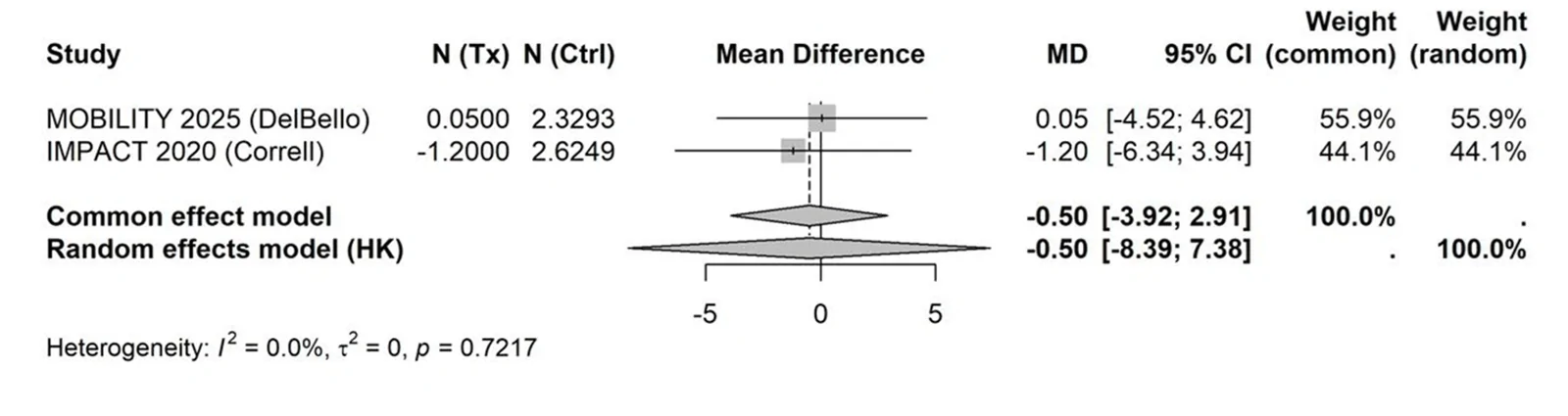
Forest plot of the effect of metformin versus control on high-density lipoprotein cholesterol (HDL-C) in children and adolescents receiving second-generation antipsychotics Forest plot showing pooled mean differences (MDs) with 95% confidence intervals (CIs) for the effect of metformin compared with control on HDL-C at medium-term follow-up (approximately 24 weeks or 6 months). Positive values indicate higher HDL-C levels in the metformin group. Squares represent study-specific effect estimates, horizontal lines represent 95% CIs, and the diamond represents the pooled random-effects estimate. Analyses were conducted using random-effects meta-analysis with restricted maximum likelihood estimation and Hartung-Knapp adjustment [4,8].

**Figure 4:**
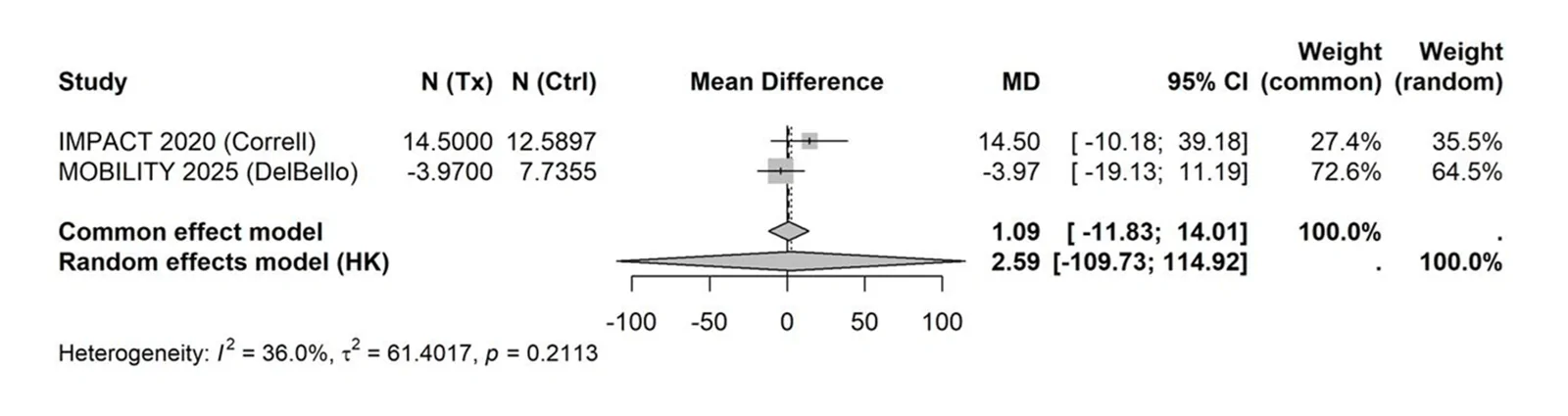
Forest plot of the effect of metformin versus control on triglyceride levels in children and adolescents receiving second-generation antipsychotics Forest plot showing pooled mean differences (MDs) with 95% confidence intervals (CIs) for the effect of metformin compared with control on triglyceride levels at medium-term follow-up (approximately 24 weeks or 6 months). Negative values indicate lower triglyceride levels in the metformin group. Squares represent study-specific effect estimates, horizontal lines represent 95% CIs, and the diamond represents the pooled random-effects estimate. Statistical analyses used a random-effects model with restricted maximum likelihood estimation and Hartung-Knapp adjustment [4,8].

Evidence for metabolic outcomes was limited. Homeostatic model assessment of insulin resistance (HOMA-IR), fasting glucose, high-density lipoprotein (HDL) cholesterol (Figure 3), and triglycerides (Figure 4) showed no statistically significant pooled differences between metformin and control groups, and estimates were imprecise. Glycated hemoglobin (HbA1c), low-density lipoprotein (LDL) cholesterol, total cholesterol, and insulin were reported in individual trials only.

Safety

Diarrhea was the most frequently reported adverse event and occurred more often with metformin, although pooled estimates were imprecise. Gastrointestinal adverse events were otherwise uncommon. Evidence regarding treatment discontinuation was inconsistent across studies, precluding meaningful pooled interpretation.

Overall, metformin consistently attenuated weight gain compared with control, but pooled improvements in BMI z-score were not statistically significant, and evidence for metabolic benefits remained inconclusive.

Prevention Versus Treatment

Only one RCT evaluated metformin for prevention of antipsychotic-associated weight gain in antipsychotic-naïve participants [1]. This trial reported significantly less weight gain and percentage weight gain with metformin; however, its high risk of bias limited confidence in these findings. Because only one prevention study was available, formal comparisons between prevention and treatment strategies were not performed.

Effects of Topiramate

One pilot RCT evaluated topiramate in adolescents receiving olanzapine [5]. Topiramate reduced increases in BMI z-score, BMI, and body weight compared with placebo. No consistent improvements were observed in metabolic laboratory measures, and discontinuation rates did not differ significantly between groups. Because evidence was limited to a single small trial with high risk of bias, conclusions remain preliminary.

Effects of Melatonin

One RCT evaluated melatonin as adjunctive therapy during olanzapine treatment [6,7]. Melatonin was associated with smaller increases in BMI and body weight, although neither outcome reached statistical significance. Limited evidence suggested possible improvements in total cholesterol and systolic blood pressure, but insufficient quantitative data were available for meta-analysis. No adverse-event data were reported. Overall, evidence for melatonin remains limited.

Effects of Antipsychotic Switching

The IMPACT trial evaluated switching from higher-risk antipsychotics to lower-risk alternatives [4]. Antipsychotic switching reduced BMI z-score and body weight at both 12 and 24 weeks compared with continued antipsychotic treatment. However, these findings were derived from a single open-label trial, and one switch subgroup was discontinued because of psychiatric worsening, highlighting potential risks associated with this strategy.

Sensitivity Analyses

Sensitivity analyses showed that exclusion of individual studies, including the large MOBILITY trial, did not change the overall direction of the pooled metformin effect. Although effect estimates consistently favored metformin, confidence intervals generally crossed the null. Heterogeneity was low for most anthropometric outcomes but moderate for short-term BMI z-score. Most secondary outcomes and all analyses of topiramate, melatonin, and antipsychotic switching were based on single trials, limiting the certainty of the evidence. Publication bias was not assessed because no outcome included 10 or more comparable studies.

Limitations

This review has several limitations. The available evidence base was small, with only seven unique randomized controlled trials and limited data contributing to each pooled analysis. Studies varied substantially in diagnosis, antipsychotic exposure, intervention timing, follow-up duration, and outcome reporting, and metabolic outcomes were often incompletely reported. No trial was judged to have a low risk of bias, and attrition and selective reporting may have influenced findings. Small-study effects could not be formally assessed, and publication bias cannot be excluded. The review relied on aggregate published data, preventing individual participant-level analyses, and the limited number of comparable studies precluded network meta-analysis. Despite these limitations, the review used rigorous methods, including restriction to randomized evidence, careful handling of companion publications and shared control groups, separation of prevention and treatment strategies, and sensitivity analyses to evaluate the robustness of findings.

## Conclusions

In children and adolescents treated with second-generation antipsychotics, metformin showed the most developed evidence base among the evaluated metabolic-protective strategies and generally reduced the extent of weight gain compared with control. However, the overall effects on standardized weight outcomes were limited, and the available evidence does not support universal use of metformin or other metabolic interventions for all patients. Evidence for alternative strategies, including topiramate, melatonin, and antipsychotic switching, remains insufficient to establish comparative effectiveness. Clinical decisions should be individualized according to patient characteristics, treatment goals, and risk-benefit considerations. Further well-designed pediatric trials with longer follow-up and standardized outcomes are needed to clarify the role of metabolic-protective strategies in routine care.

## Figures and Tables

**Figure 5:**
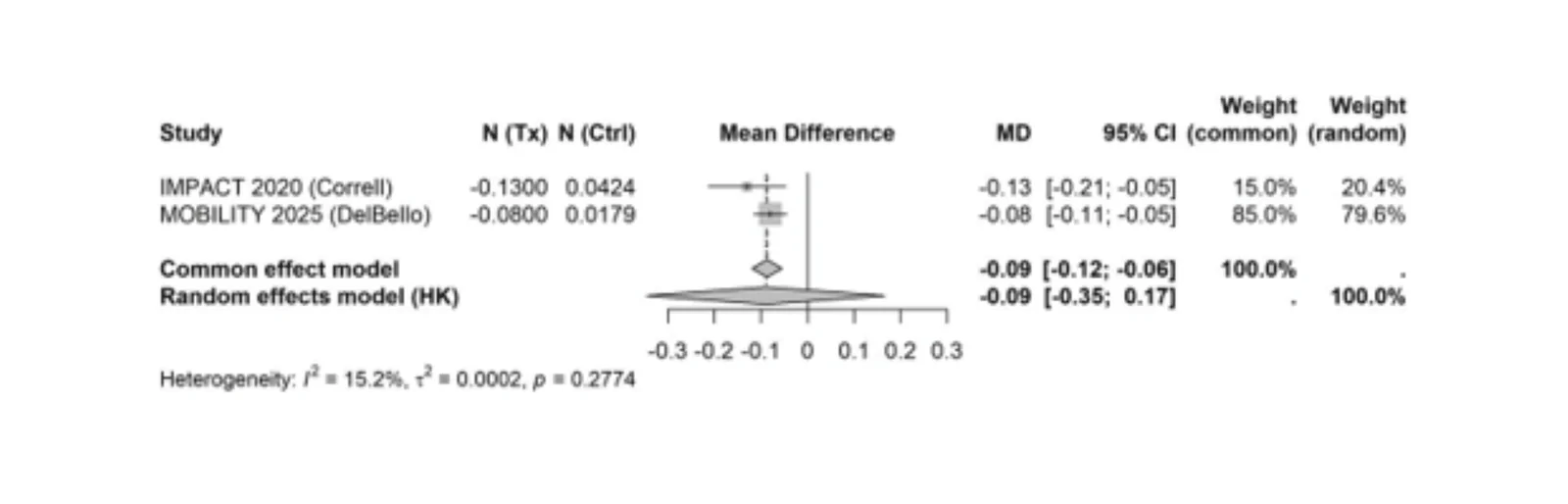
Forest plot, metformin vs. control, BMI z-score, medium-term

**Figure 6:**
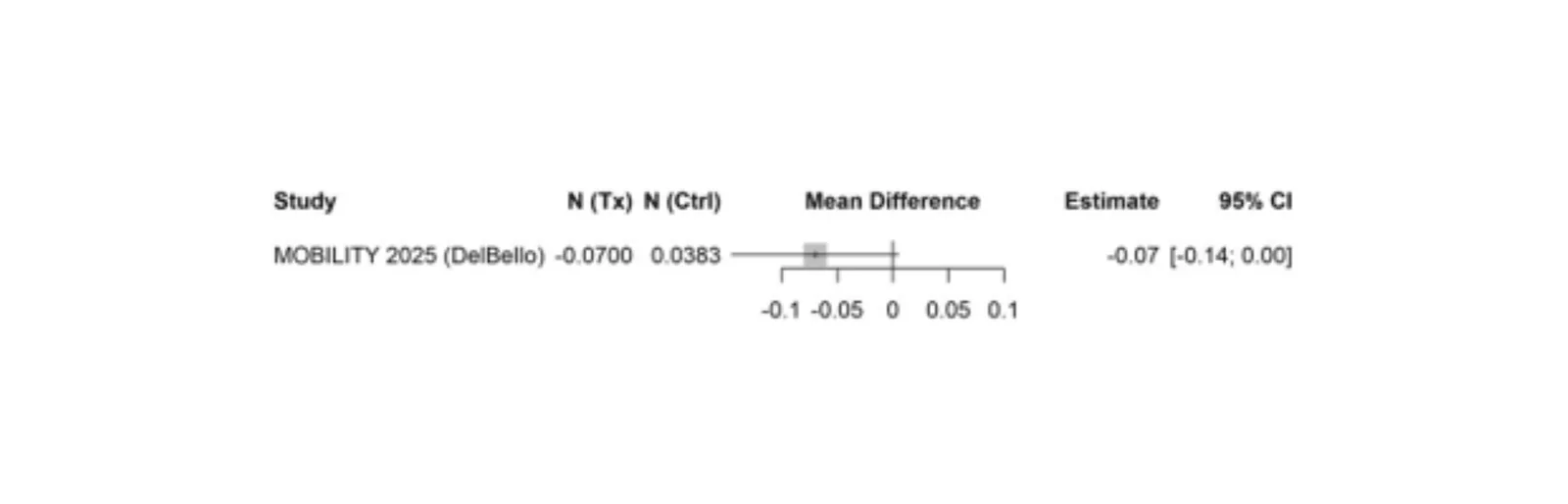
Forest plot, metformin vs. control, BMI z-score, long-term (single trial, not pooled)

## Appendices

Figures 5-6 show the medium-term and long-term analyses of the body mass index z-score.
